# Alterations in circulating lipidomic profile in patients with type 2 diabetes with or without non-alcoholic fatty liver disease

**DOI:** 10.3389/fmolb.2023.1030661

**Published:** 2023-02-24

**Authors:** Assim A. Alfadda, Abdulrahman M. Almaghamsi, Suphia Murad Sherbeeni, Adel N. Alqutub, Abdullah S. Aldosary, Arthur C. Isnani, Nasser Al-Daghri, Simon D. Taylor-Robinson, Rukhsana Gul

**Affiliations:** ^1^ Obesity Research Center, College of Medicine, King Saud University, Riyadh, Saudi Arabia; ^2^ Department of Medicine, College of Medicine, King Saud University, Riyadh, Saudi Arabia; ^3^ Obesity, Endocrine, and Metabolism Center, King Fahd Medical City, Riyadh, Saudi Arabia; ^4^ Tadaw Medical Complex and Day Surgery Center, Riyadh, Saudi Arabia; ^5^ Department of Gastroenterology and Hepatology, King Fahad Medical City, Riyadh, Saudi Arabia; ^6^ Department of Medical Imaging Administration, King Fahad Medical City, Riyadh, Saudi Arabia; ^7^ Chair for Biomarkers of Chronic Diseases, Department of Biochemistry, College of Science, King Saud University, Riyadh, Saudi Arabia; ^8^ Department of Surgery and Cancer, St. Mary’s Hospital Campus, Imperial College London, London, United Kingdom

**Keywords:** nuclear—magnetic resonance, metabolomics, non-alcoholic fatty liver (NAFLD), diabetes, lipidomics

## Abstract

**Objective:** Non-alcoholic fatty liver disease (NAFLD) and Type 2 diabetes mellitus (T2DM) often coexist and drive detrimental effects in a synergistic manner. This study was designed to understand the changes in circulating lipid and lipoprotein metabolism in patients with T2DM with or without NAFLD.

**Methods:** Four hundred thirty-four T2DM patients aged 18–60 years were included in this study. Fatty liver was assessed by FibroScan. The comprehensive metabolic lipid profiling of serum samples was assessed by using high-throughput proton NMR metabolomics.

**Results:** Our data revealed a significant association between steatosis and serum total lipids in VLDL and LDL lipoprotein subclasses, while total lipids in HDL subclasses were negatively associated. A significant positive association was found between steatosis and concentration of lipids, phospholipids, cholesterol, and triglycerides in VLDL and LDL subclasses, while HDL subclasses were negatively associated. Furthermore, a significant, association was observed between fibrosis and concentrations of lipids, phospholipids, cholesterol, and triglycerides in very small VLDL, large, and very large HDL subclasses. Subgroup analysis revealed a decrease in the concentrations of lipids, phospholipids, cholesterol, and other lipid biomolecules in patients using antilipemic medications.

**Conclusion:** The metabolomics results provide evidence that patients with T2DM with higher steatosis grades have altered lipid metabolomics compared to patients without steatosis. Increased lipid, phospholipids, cholesterol, and triglycerides concentration of VLDL and LDL subclasses are associated with steatosis in patients with T2DM.

## Introduction

Non-alcoholic fatty liver disease (NAFLD) is the most common hepatic condition detected in patients with Type 2 diabetes (T2DM) with an estimated prevalence of 65% among the Saudi population (Alfadda et al., 2022). The fatty liver primarily known as steatosis is defined by the presence of >5% of fat infiltration in hepatocytes (Contos and Sanyal, 2002). Fatty liver can progress to a more severe condition non-alcoholic steatohepatitis (NASH) causing hepatocyte inflammation which can finally progress to permanent end stage liver disease such as cirrhosis and hepatocellular carcinoma (Angulo et al., 1999).

Steatosis or fatty liver is linked with dyslipidemia due to the increased synthesis and deposition of lipids in the hepatocytes which attribute to the formation of lipid droplets (Cohen et al., 2011). This increased intrahepatic fat accumulation causes lipotoxicity, comorbidity associated with fatty liver resulting from an increase in the levels of fatty acids in the serum that flow from peripheral adipose tissues to the liver (Wasilewska and Lebensztejn, 2021). The increased fat accumulation in fatty liver is a result of the imbalance in fatty acid uptake, increased production of lipids *via* hepatic *de novo* lipogenesis, inadequate hepatic fat export through very‐low‐density lipoproteins (VLDLs) and its oxidation of fatty acids (Dowman et al., 2010). An increase in lipotoxicity in NAFLD due to impaired fatty acid oxidation activate inflammatory signaling that attributes to hepatocyte injury and are accountable for the progression to NASH (Trovato et al., 2014).

We have recently revealed the prevalence NAFLD in a Saudi cohort of patients with T2DM and demonstrated the associations between fatty liver and dyslipidemia (Alfadda et al., 2022). Our data showed that controlled attenuation parameter (CAP) values are positively correlated with triglycerides, and negatively with high-density lipoprotein (HDL). Given the importance of serum lipoproteins in NAFLD, here we aimed to investigate the alterations in serum lipids and lipoprotein subclasses using high-throughput proton nuclear magnetic resonance (NMR) spectroscopy metabolomics in patients with T2DM with or without steatosis. Recent studies have expanded knowledge regarding the lipid profiling in fatty liver and revealed the associations between serum lipidome and NAFLD (Kaikkonen et al., 2017). However, to the best of our knowledge no studies have been conducted using NMR-metabolomic approach to investigate the changes in circulating lipids and lipoproteins in Saudi patients with T2DM with or without NAFLD.

## Methods

### Study population

This study included patients who participated in the Cohort of Non-alcoholic Fatty Liver Disease in Saudis with T2DM (the CORDIAL Study). This prospective cohort study started in 2015 and recruited patients from King Fahad Medical City (KFMC) and affiliated Primary Care Centers in Riyadh, Saudi Arabia. The cohort was approved by the Institutional Review Board at KFMC (study number: 12–344), and all patients provided written, informed consent prior to recruitment. The study was conducted in accordance with the ethical principles for medical research on human subjects adopted by the 18th World Medical Association General Assembly, and the Declaration of Helsinki 1964 and its subsequent amendments. The inclusion criteria included Saudi patients aged 18–60 years who were diagnosed with T2DM and followed up regularly in the diabetes or primary care clinics. Patients were excluded if they tested positive for hepatitis B surface antigen or had antibodies against hepatitis C virus, were diagnosed with other chronic liver diseases (e.g., hemochromatosis, primary biliary cholangitis, or autoimmune hepatitis), known to have pre-existing hepatic or extrahepatic malignancy, or were consuming >20 g of alcohol per day. The patients will be prospectively followed for 10 years and assessed for hepatic, metabolic, renal, and cardiovascular complications.

### Sample size calculation

Using sampling formula for a single cross-sectional survey: Sample size = Z_1−α/2_^2^ *p* (1−p)/d^2^, where Z1−a/2 = is the standard normal variate (at 5% type 1 error (*p* < 0.05) or 1% type 1 error (*p* < 0.01). As in majority of studies *p* values are considered significant below 0.05 hence 1.96 is used in formula, *p* = expected proportion in population and d = the absolute error or precision. Based on 95% confidence interval (1.96) and absolute precision of 5%. Using a previously reported estimated all ages prevalence of NAFLD of 24.8% in Saudi Arabia (Alswat et al., 2018), the calculated sample size was 287. To account for loss of cases, it was decided to include 434 patients.

### Clinical and laboratory data collection

The participants’ characteristics and anthropometric indices, including age, sex, body weight, height, body mass index (BMI), and blood pressure, were obtained. BMI was calculated as body weight (kg) divided by body height (m2). Blood was sampled for laboratory assays after the patients had fasted for ten to 12 h overnight. Fasting blood glucose and serum lipids were measured using Abbott—Architect Plus, a clinical chemistry autoanalyzer (Abbott, Abbott Park, IL, United States). Glycated hemoglobin (HbA1c) determination was performed using D-100®, a high-performance liquid chromatography analyzer (Bio-Rad Laboratories, Hercules, CA, United States).

### Liver FibroScan examination

FibroScan® 502 and FibroScan® 530 Compact, with two probes - Medium (M+) and Extra-large (XL+) (Echosens Ltd., Paris, France) were used for measuring CAP—as surrogate measure of liver fat content, and liver stiffness measurement (LSM)—as a surrogate measure of hepatic fibrosis. The device estimates liver steatosis in decibel/meter (dB/m) and liver stiffness in kilopascal (kPa). CAP and LSM were obtained simultaneously in each examination. The type of probe required for each participant was selected by an automatic probe selection tool embedded within the FibroScan® operating software. A successful vibration controlled transient elastography (VCTE) exam was defined by the acquisition of ten successful measurements, where the interquartile range of the LSM did not exceed 30% of the median LSM. Therefore, an “uninterpretable” VCTE examination encompassed failures on one or both accounts. Each patient underwent VCTE examination after 3 h of fasting. All VCTE examinations were performed by two experienced physicians. The optimal cut-off values for classifying steatosis grades were as follows: S0 (CAP <248 dB/m), no steatosis; S1 (CAP 248 to <268 dB/m), mild steatosis; S2 (CAP 268 to <280 dB/m), moderate steatosis; and (S3 CAP ≥280 dB/m), severe steatosis (Karlas et al., 2017), and the optimal cut-off values for classifying fibrosis grades were: F0-F1 (<7.9 kPa), no fibrosis; F2 (7.9 to <8.8 kPa), moderate fibrosis; F3 (8.8 to <11.7 kPa), severe fibrosis; and F4 (≥11.7 kPa), liver cirrhosis (Abeysekera et al., 2020).

### Quantitative NMR metabolic profiling

Metabolic biomarkers were quantified from serum samples using untargeted high-throughput proton nuclear magnetic resonance (NMR) spectroscopy metabolomics platform (Nightingale Health Plc, Helsinki, Finland). The details of the methodology used have been described previously (Soininen et al., 2015). The samples were barcoded for sample identification and kept frozen at −80°C for analysis. Metabolites were measured by a quantitative high-throughput NMR experimental set up for the simultaneous quantification of lipids and lipoprotein subclass profiling in 350 µL of serum. All liquid handling procedures were completed prior to the NMR studies, and the SampleJet robotic sample charger was set up at a cooled temperature to prevent sample deterioration. Every single metabolic measurement was subjected to a number of statistical quality control procedures and cross-referenced with a sizable collection of quantitative molecular data.

### Statistical analysis

Data were analyzed using the Statistical Package for Social Sciences (SPSS) version 23.0 (SPSS Inc., IBM, Armonk, New York, United States). Test of normality of distribution was carried out using the Shapiro-Wilk test. The results were expressed as numbers and percentages (categorical variables) and as mean, standard deviation, and minimum and maximum for continuous variables. Measurement of the strength and direction of the relationship/correlation between two continuous and categorical variables in normally distributed data was performed using the Pearson correlation test and the chi-square (X2) test, respectively. An independent student t-test was performed to determine the difference between two means. One-way analysis of variance (ANOVA) with Tukey’s *post hoc* analysis was used to determine significant differences in the means of the laboratory results according to grades of steatosis. All *p*-values were two-tailed, and statistical significance was set at *p* < 0.05.

## Results

### Demographic and baseline laboratory characteristic of the patients

**FIGURE 1:**
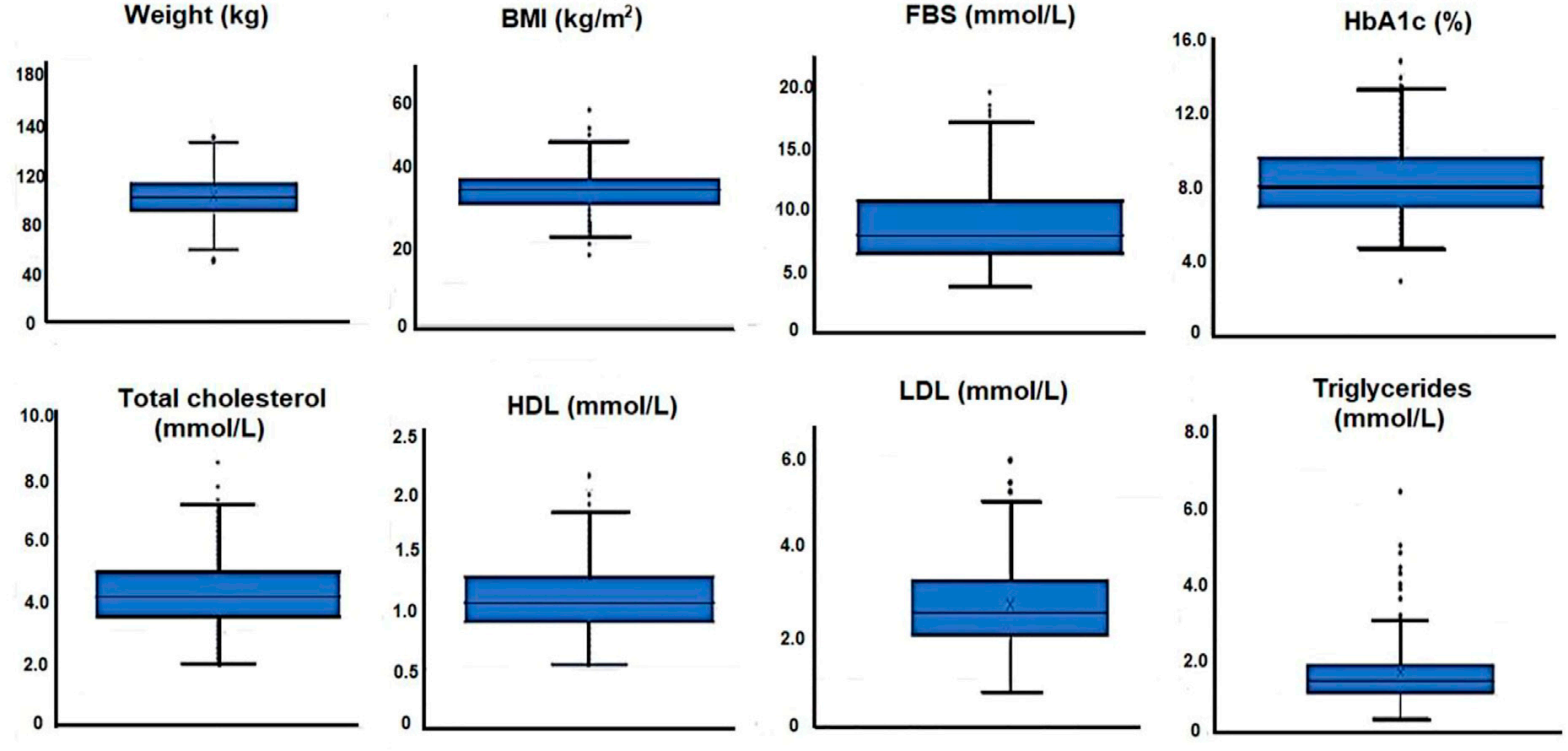
Box and whisker plots of baseline anthropometrics and laboratory tests for 434 patients with T2DM.

In total, 434 patients aged 18–60 years diagnosed with T2DM (227, 52.3% males and 207, 47.7% females) were included in this analysis. The mean age of all patients was 50.1 ± 7.6 years. The mean BMI was 32.64 ± 5.7 kg/m^2^, where 287 patients (66.1%) were diagnosed with obesity (BMI ≥30 kg/m^2^). The duration of diabetes ranged from 1 to 41 years (mean = 10.66 ± 7.5 years). Figure 1 shows the box and whisker plots of baseline anthropometrics and laboratory tests.

In terms of medication use 217 (50%) were on oral hypoglycemic agents mainly Metformin, 80 (18.4%) were on insulin, 161 (37.1%) were on antihypertensive and 203 (46.7%) were on antilipemic medication.

FibroScan showed out of 434 patients the proportion of patients with no steatosis (S0), mild (S1), moderate (S2) and severe steatosis (S3) were 89 (20.5%) (CAP <248 dB/m), 41 (9.4%) (CAP≥248 to <268 dB/m), 24 (5.5%) (CAP≥268 to <280 dB/m), 280 (64.5%) (CAP≥280 dB/m) respectively. Conversely, the proportion of patients without (F0-F1) or with (F2-F4) fibrosis were 398 (91.7%) (LSM<7.9 kPa) and 36 (8.2%) (LSM≥7.9 kPa) respectively. Supplementary Table S1 shows the lipidomic profile for all 434 patients with T2DM. A total of 81 lipid types, species and biomolecules were identified and analyzed. These included total lipids in lipoprotein particles, total phospholipids, total cholesterol and triglycerides in lipoprotein particles, phosphoglycerides, total cholines, phosphatidylcholines, sphingomyelins, apolipoproteins B and A1, and fatty acids. Lipidomic profile for all patients are shown in Supplementary Table S1.

### Association between liver steatosis and lipid biomolecules

Table 1 shows the association between lipid biomolecules and steatosis using the CAP values measured in dB/m. Steatosis was positively associated with total lipids in lipoprotein particles (*r* = 0.113, *p* = 0.018), including total lipids in chylomicrons and extremely large VLDL, and total lipids in very large, large, medium and small size VLDL particles (*r* = 0.211, *p* < 0.001, *r* = 0.225, *p* < 0.001, *r* = 0.224, *p* < 0.001, *r* = 0.183, *p* < 0.001, and *r* = 0.152, *p* = 0.001, respectively). Total lipids in medium LDL was also positively associated with CAP (*r* = 0.094, *p* = 0.050). Total lipids in very large HDL and large HDL were negatively associated with CAP (*r* = −0.239, *p* < 0.001 and *r* = −0.207, *p* < 0.001, respectively), while total lipids in small HDL was positively associated with CAP (*r* = 0.189, *p* = ˂0.001). For phospholipids in lipoprotein particles, CAP was positively associated with phospholipids in chylomicrons and extremely large VLDL, and large, medium, and small VLDL (*r* = 0.204, *p* < 0.001, *r* = 0.221, *p* < 0.001, *r* = 0.222, *p* < 0.001, *r* = 0.150, *p* = 0.002, *r* = 0.117, *p* = 0.014). On the other hand, phospholipids in very large, large and small HDL were negatively associated with CAP (*r* = −0.232, *p* < 0.001, *r* = −0.191, *p* < 0.001, *r* = −0.175, *p* < 0.001, respectively). Positive association was observed between CAP and cholesterol in chylomicrons and extremely large VLDL, very large VLDL, and large VLDL (*r* = 0.213, *p* < 0.001, *r* = 0.209, *p* < 0.001, and r = 0.208, *p* < 0.001, respectively). On the other hand, negative associations were observed between CAP and cholesterol in HDL, very large HDL, large HDL and small HDL (*r* = −0.124, *p* = 0.010, *r* = −0.256, *p* < 0.001, *r* = −0.229, *p* < 0.001, *r* = −0.160, *p* = 0.001, respectively). Triglycerides and triglycerides in chylomicrons and extremely large to small subclasses of VLDL, and medium to small subclasses of LDL were positively associated with CAP (*p* < 0.05). The ratio of triglycerides to phosphoglycerides was positively associated with CAP (*r* = 0.255, *p* < 0.001). Other lipid biomolecules that were found to be associated with CAP were total fatty acids, omega 3 and omega 6 fatty acids, polyunsaturated fatty acids (PUFA), monounsaturated fatty acids (MUFA) and saturated fatty acids (SFA) (*r* = 0.185, *p* < 0.001, *r* = 0.119, *p* = 0.014, *r* = 0.100, *p* = 0.038, *r* = 0.111, *p* = 0.021, *r* = 0.196, *p* < 0.001 and *r* = 0.211, *p* < 0.001, respectively).

**TABLE 1:** Association of lipid biomolecules to steatosis, and lipid biomolecules according to steatosis grades in patients with T2DM.

Lipid biomolecules	Association with steatosis CAP (dB/m) *r* (*p*-value)	S0 CAP <248 dB/m	S1 CAP 248 to <268 dB/m	S2 CAP 268 to <280 dB/m	S3 CAP ≥280 dB/m	ANOVA *p* values	Post-hoc analysis (*p*-value)
		*N* = 89	*N* = 41	*N* = 24	*N* = 280		
Total lipids in lipoprotein particles	0.113 (0.018)	9.36 (2.1)	9.09 (1.9)	9.30 (1.7)	9.60 (2.2)	0.432	S0-S3 (0.001)
Total lipids in chylomicrons extremely large VLDL	0.211 (<0.001)	0.200 (0.23)	0.093 (0.13)	0.389 (0.50)	0.233 (0.20)	0.001	S0-S3 (0.001)
Total lipids in very large VLDL	0.225 (<0.001)	0.21 (0.14)	0.22 (0.18)	0.27 (0.21)	0.31 (0.23)	0.001	S0-S3 (0.001)
Total lipids in large VLDL	0.224 (<0.001)	0.36 (0.2)	0.37 (0.2)	0.43 (0.3)	0.49 (0.3)	0.001	S0-S3 (0.002)
							S1/S3 (0.044)
Total lipids in medium VLDL	0.183 (<0.001)	0.63 (0.2)	0.57 (0.3)	0.64 (0.2)	0.71 (0.2)	0.006	S1-S3 (0.016)
Total lipids in small VLDL	0.152 (0.001)	0.439 (0.2)	0.416 (0.1)	0.428 (0.1)	0.473 (0.1)	0.046	S1-S3 (0.038)
Total lipids in very small VLDL	0.017 (0.731)	0.369 (0.12)	0.350 (0.09)	0.333 (0.07)	0.362 (0.10)	0.470	NS
Total lipids in IDL	−0.052 (0.278)	1.26 (0.4)	1.17 (0.3)	1.15 (0.3)	1.17 (0.3)	0.141	NS
Total lipids in large LDL	0.019 (0.698)	1.78 (0.4)	1.66 (0.4)	1.67 (0.4)	1.72 (0.4)	0.392	NS
Total lipids in medium LDL	0.094 (0.050)	0.778 (0.2)	0.723 (0.2)	0.747 (0.2)	0.780 (0.2)	0.329	NS
Total lipids in small LDL	0.082 (0.088)	0.349 (0.1)	0.325 (0.1)	0.338 (0.1)	0.349 (0.1)	0.309	NS
Total lipids in HDL	−0.067 (0.166)	3.01 (0.6)	3.08 (0.5)	3.04 (0.6)	2.93 (0.5)	0.196	NS
Total lipids in very large HDL	−0.239 (<0.001)	0.132 (0.07)	0.125 (0.04)	0.118 (0.05)	0.105 (0.05)	0.001	S0-S3 (0.001)
Total lipids in large HDL	−0.207 (<0.001)	0.55 (0.2)	0.56 (0.1)	0.53 (0.3)	0.46 (0.2)	0.001	S0-S3 (0.004)
							S1-S3 (0.041)
Total lipids in medium HDL	−0.002 (0.964)	1.05 (0.2)	1.09 (0.2)	1.08 (0.2)	1.05 (0.2)	0.436	NS
Total lipids in small HDL	0.189 (<0.001)	1.27 (0.2)	1.29 (0.2)	1.31 (0.2)	1.32 (0.2)	0.128	NS
***Total phospholipids in lipoprotein particles***	0.066 (0.172)	2.95 (0.6)	2.92 (0.5)	2.93 (0.5)	2.96 (0.5)	0.95	NS
Phospholipids in chylomicrons and extremely large VLDL	0.204 (<0.001)	0.023 (0.02)	0.010 (0.02)	0.048 (0.06)	0.027 (0.025)	0.001	S0-S3 (0.002)
Phospholipids in very large VLDL	0.221 (<0.001)	0.036 (0.03)	0.037 (0.03)	0.045 (0.04)	0.053 (0.04)	0.001	S0-S3 (0.002)
Phospholipids in large VLDL	0.222 (<0.001)	0.067 (0.04)	0.067 (0.05)	0.079 (0.05)	0.092 (0.06)	0.001	S0-S3 (0.002)
							S1-S3 (0.041)
Phospholipids in medium VLDL	0.150 (0.002)	0.133 (0.05)	0.119 (0.05)	0.131 (0.04)	0.143 (0.05)	0.033	S1-S3 (0.037)
Phospholipids in small VLDL	0.117 (0.014)	0.101 (0.03)	0.094 (0.03)	0.096 (0.03)	0.105 (0.03)	0.147	NS
Phospholipids in very small VLDL	0.028 (0.560)	0.100 (0.03)	0.095 (0.02)	0.091 (0.02)	0.099 (0.03)	0.46	NS
Phospholipids in IDL	−0.049 (0.308)	0.303 (0.08)	0.285 (0.06)	0.278 (0.08)	0.284 (0.07)	0.156	NS
Phospholipids in large LDL	0.013 (0.780)	0.40 (0.1)	0.38 (0.1)	0.38 (0.1)	0.38 (0.1)	0.388	NS
Phospholipids in medium LDL	0.085 (0.075)	0.191 (0.05)	0.178 (0.04)	0.83 (0.04)	0.190 (0.04)	0.415	NS
Phospholipids in small LDL	0.053 (0.268)	0.097 (0.02)	0.090 (0.02)	0.093 (0.02)	0.096 (0.02)	0.314	NS
Phospholipids in HDL	−0.040 (0.407)	1.50 (0.3)	1.55 (0.2)	1.52 (0.3)	1.48 (0.3)	0.355	NS
Phospholipids in very large HDL	−0.232 (<0.001)	0.054 (0.04)	0.051 (0.02)	0.046 (0.03)	0.039 (0.03)	0.002	S0-S3 (0.002)
Phospholipids in large HDL	−0.191 (<0.001)	0.28 (0.1)	0.28 (0.1)	0.26 (0.1)	0.22 (0.1)	0.003	S0-S3 (0.016)
							S1-S3 (0.039)
Phospholipids in medium HDL	−0.031 (0.518)	0.48 (0.1)	0.50 (0.1)	0.49 (0.1)	0.48 (0.1)	0.497	NS
Phospholipids in small HDL	−0.175 (<0.001)	0.70 (0.1)	0.72 (0.1)	0.72 (0.1)	0.73 (0.1)	0.151	NS
***Total cholesterol***	0.019 (0.688)	5.14 (1.3)	4.87 (1.1)	4.91 (1.1)	5.0 (1.1)	0.555	NS
Cholesterol in chylomicrons and extremely large VLDL	0.213 (<0.001)	0.045 (0.05)	0.019 (0.03)	0.07 (0.09)	0.05 (0.04)	0.001	S0-S3 (0.001)
Cholesterol in very large VLDL	0.209 (<0.001)	0.061 (0.03)	0.060 (0.04)	0.07 (0.04)	0.08 (0.05)	0.002	S0-S3 (0.006)
							S1-S3 (0.043)
Cholesterol in large VLDL	0.208 (<0.001)	0.102 (0.06)	0.108 (0.06)	0.120 (0.07)	0.140 (0.08)	0.002	S0-S3 (0.007)
							S1-S3 (0.037)
Cholesterol in medium VLDL	0.037 (0.436)	0.203 (0.08)	0.177 (0.08)	0.183 (0.07)	0.196 (0.08)	0.297	NS
Cholesterol in small VLDL	0.084 (0.082)	0.190 (0.07)	0.174 (0.06)	0.173 (0.05)	0.192 (0.06)	0.217	NS
Cholesterol in very small VLDL	−0.034 (0.476)	0.203 (0.06)	0.190 (0.06)	0.179 (0.05)	0.192 (0.06)	0.242	NS
Cholesterol in IDL	−0.066 (0.168)	0.86 (0.2)	0.79 (0.2)	0.77 (0.2)	0.77 (0.2)	0.089	NS
Cholesterol in large LDL	0.014 (0.768)	1.29 (0.4)	1.19 (0.3)	1.20 (0.3)	1.24 (0.3)	0.371	NS
Cholesterol in medium LDL	0.091 (0.057)	0.555 (0.2)	0.513 (0.1)	0.531 (0.1)	0.556 (0.1)	0.325	NS
Cholesterol in small LDL	0.078 (0.106)	0.238 (0.06)	0.221 (0.06)	0.229 (0.05)	0.237 (0.06)	0.323	NS
Cholesterol in HDL	−0.124 (0.010)	1.39 (0.3)	1.40 (0.2)	1.39 (0.3)	1.32 (0.2)	0.017	NS
Cholesterol in very large HDL	−0.256 (<0.001)	0.07 (0.03)	0.07 (0.02)	0.06 (0.02)	0.05 (0.02)	<0.0001	S0-S3 (<0.001)
Cholesterol in large HDL	−0.229 (<0.001)	0.26 (0.1)	0.26 (0.1)	0.24 (0.1)	0.20 (0.1)	<0.0001	S0-S3 (<0.001)
							S1-S3 (0.037)
Cholesterol in medium HDL	−0.059 (0.219)	0.53 (0.1)	0.54 (0.1)	0.54 (0.1)	0.51 (0.1)	0.124	NS
Cholesterol in small HDL	−0.160 (0.001)	0.53 (0.1)	0.53 (0.1)	0.54 (0.1)	0.54 (0.1)	0.452	NS
***Triglycerides***	0.218 (<0.001)	1.27 (0.6)	1.30 (0.7)	1.46 (0.8)	1.64 (0.9)	0.001	S0-S3 (0.001)
Triglycerides in chylomicrons and extremely large VLDL	0.212 (<0.001)	0.132 (0.15)	0.063 (0.08)	0.265 (0.345)	0.155 (0.135)	0.001	S0-S3 (0.001)
Triglycerides in very large VLDL	0.230 (<0.001)	0.117 (0.08)	0.124 (0.12)	0.153 (0.13)	0.177 (0.14)	<0.0001	S0-S3 (0.001)
Triglycerides in large VLDL	0.229 (<0.001)	0.186 (0.11)	0.193 (0.14)	0.226 (0.15)	0.260 (0.17)	<0.0001	S0-S3 (0.001)
Triglycerides in medium VLDL	0.227 (<0.001)	0.293 (0.13)	0.273 (0.13)	0.324 (0.16)	0.368 (0.19)	<0.0001	S0-S3 (0.002)
Triglycerides in small VLDL	0.203 (<0.001)	0.148 (0.06)	0.148 (0.06)	0.158 (0.06)	0.177 (0.08)	0.002	S0-S3 (0.005)
Triglycerides in very small VLDL	0.134 (0.005)	0.065 (0.02)	0.064 (0.02)	0.064 (0.02)	0.070 (0.02)	0.079	NS
Triglycerides in IDL	0.089 (0.065)	0.97 (0.02)	0.095 (0.02)	0.094 (0.02)	0.101 (0.02)	0.35	NS
Triglycerides in large LDL	0.087 (0.069)	0.097 (0.02)	0.094 (0.02)	0.094 (0.02)	0.100 (0.02)	0.373	NS
Triglycerides in medium LDL	0.138 (0.004)	0.032 (0.01)	0.031 (0.01)	0.032 (0.01)	0.034 (0.01)	0.094	NS
Triglycerides in small LDL	0.184 (<0.001)	0.013 (0.01)	0.013 (0.01)	0.015 (0.01)	0.016 (0.01)	0.009	S0-S3 (0.021)
Triglycerides in HDL	0.153 (0.001)	0.12 (0.04)	0.12 (0.04)	0.12 (0.03)	0.13 (0.05)	0.037	S0-S3 (0.030)
Triglycerides in very large HDL	−0.211 (<0.001)	0.005 (0.01)	0.005 (0.01)	0.006 (0.01)	0.006 (0.01)	0.775	NS
Triglycerides in large HDL	−0.010 (0.842)	0.02 (0.01)	0.02 (0.01)	0.02 (0.01)	0.02 (0.01)	0.916	NS
Triglycerides in medium HDL	0.166 (<0.001)	0.043 (0.01)	0.047 (0.02)	0.046 (0.01)	0.050 (0.01)	0.019	S0-S3 *p* = 0.012
Triglycerides in small HDL	0.227 (<0.001)	0.048 (0.01)	0.050 (0.01)	0.051 (0.01)	0.056 (0.01)	0.001	S0-S3 (0.001)
Phosphoglycerides	0.111 (0.021)	2.55 (0.53)	2.53 (0.49)	2.52 (0.42)	2.60 (0.51)	0.643	NS
Ratio of triglycerides to phosphoglycerides	0.255 (<0.001)	0.49 (0.17)	0.50 (0.21)	0.58 (0.30)	0.60 (0.22)	<0.0001	S0-S3 (<0.001)
							S1-S3 (0.020)
Total cholines	0.087 (0.070)	2.85 (0.55)	2.81 (0.48)	2.81 (0.44)	2.88 (0.50)	0.736	NS
Phosphatidylcholines	−0.104 (0.031)	2.34 (0.50)	2.32 (0.48)	2.30 (0.41)	2.39 (0.41)	0.583	NS
Sphingomyelins	−0.035 (0.466)	0.54 (0.1)	0.51 (0.07)	0.51 (0.09)	0.52 (0.08)	0.172	NS
Apolipoprotein B	0.052 (0.281)	0.98 (0.31)	0.90 (0.27)	0.91 (0.25)	0.96 (0.27)	0.404	NS
Apolipoprotein A1	−0.027 (0.573)	1.38 (0.26)	1.42 (0.22)	1.40 (0.24)	1.36 (0.21)	0.45	NS
Ratio of apolipoprotein B to apolipoprotein A1	0.067 (0.162)	0.72 (0.22)	0.64 (0.20)	0.70 (0.16)	0.71 (0.20)	0.109	NS
Total fatty acids	0.185 (<0.001)	13.14 (2.3)	13.04 (2.4)	13.36 (2.1)	13.87 (2.7)	0.044	NS
Degree of unsaturation	−0.035 (0.470)	1.32 (0.08)	1.29 (0.09)	1.29 (0.10)	1.30 (0.09)	0.270	NS
Omega-3 fatty acids	0.119 (0.014)	0.52 (0.14)	0.52 (0.19)	0.47 (0.14)	0.54 (0.16)	0.168	NS
Omega-6 fatty acids	0.100 (0.038)	4.80 (0.72)	4.70 (0.66)	4.78 (0.59)	4.86 (0.71)	0.556	NS
Polyunsaturated fatty acids	0.111 (0.021)	5.32 (0.8)	5.22 (0.8)	5.25 (0.7)	5.39 (0.8)	0.492	NS
Monounsaturated fatty acids	0.196 (<0.001)	3.33 (0.7)	3.29 (0.8)	3.52 (0.8)	3.64 (0.9)	0.008	S0-S3 (0.021)
Saturated fatty acids	0.211 (<0.001)	4.49 (0.9)	4.53 (0.9)	4.59 (0.8)	4.83 (1.0)	0.016	S0-S3 (0.024)
Linoleic acid	0.092 (0.057)	3.70 (0.8)	3.62 (0.7)	3.72 (0.7)	3.76 (0.8)	0.759	NS
Docosahexaenoic acid	0.006 (0.895)	0.24 (0.06)	0.24 (0.07)	0.22 (0.05)	0.24 (0.05)	0.208	NS

VLDL, very low density lipoprotein; IDL, intermediate density lipoprotein; LDL, low density lipoprotein; HDL, high density lipoprotein; CAP, controlled attenuation parameter, S0—no steatosis; S1—mild steatosis; S2—moderate steatosis; S3—severe steatosis; NS, not significant.

The analysis of variance (ANOVA) with *post hoc* analysis showed significant differences between stage 0 and stage 3 steatosis in several lipid biomolecules. Patients with stage 3 steatosis had significantly higher total lipids in lipoprotein particles, total lipids in chylomicrons and extremely large VLDL, total lipids in very large, large, and medium size VLDL particles, and total lipids in very large and large HDL particles. Furthermore, patients with stage 3 steatosis had higher phospholipids, cholesterol and triglycerides in several lipoprotein particles. Their ratio of triglycerides to phosphoglycerides, and their mean MUFA and SFA were significantly higher when compared to patients with no steatosis (Table 1).

### Association between liver fibrosis and lipid biomolecules

We observed several significant but weak positive associations between liver fibrosis, assessed by measuring liver stiffness in kPa, and lipid biomolecules including total lipids in very small VLDL (*r* = 0.135, *p* = 0.005), total lipids in very large (*r* = 0.278, *p* < 0.001), and large (*r* = 0.199, *p* < 0.001) HDL, phospholipids in very small VLDL (*r* = 0.143, *p* = 0.003), and phospholipids in HDL (*r* = 0.104, *p* = 0.031), very large (*r* = 0.288, *p* < 0.001), and large (*r* = 0.200, *p* < 0.001) HDL. Positive association was observed between liver stiffness and cholesterol in very small VLDL (*r* = 0.129, *p* = 0.007), and very large (*r* = 0.253, *p* < 0.001), and large (*r* = 0.186, *p* < 0.001) HDL, and triglycerides in very small VLDL (*r* = 0.101, *p* = 0.035), IDL (*r* = 0.134, *p* = 0.005), large LDL (*r* = 0.126, *p* = 0.008), HDL (*r* = 0.100, *p* = 0.037), very large HDL (*r* = 0.146, *p* = 0.002) and large HDL (*r* = 0.217, *p* < 0.001). On the other hand, we observed a significantly negative correlation between liver stiffness and total lipids in small HDL (*r* = −0.109, *p* = 0.023), and cholesterol in small HDL (*r* = -0.165, *p* = 0.001). Compared to patients without fibrosis, patients with fibrosis had significantly higher mean total lipids in very small VLDL (*p* = 0.011), phospholipids in very small VLDL (*p* = 0.007) and cholesterol in very small VLDL (*p* = 0.016). Furthermore, patients with fibrosis also had significantly higher mean total lipids in very large HDL and large HDL (*p* < 0.001 and *p* = 0.027), phospholipids in very large and large HDL (*p* < 0.001 and *p* = 0.027), cholesterol in very large and large HDL (*p* < 0.001 and *p* = 0.041), and triglycerides in very large and large HDL (*p* = 0.017 and *p* = 0.003). Mean triglycerides in IDL and large IDL was also significantly higher among patients with fibrosis (*p* = 0.018 and *p* = 0.026). On the other hand, mean total lipids in small HDL and mean cholesterol in small HDL were significantly lower among patients with fibrosis (*p* = 0.024 and *p* = 0.002) as shown in Table 2.

**TABLE 2:** Association of lipid biomolecules to fibrosis, and lipid biomolecules according to fibrosis grades in 434 patients with T2DM.

Biomolecules	Fibrosis E (Kpa) correlation coefficient (*p*-value)	Without fibrosis *N* = 398	With fibrosis *N* = 36	*p*-value
Total lipids in lipoprotein particles	0.010 (0.828)	9.48 (2.1)	9.55 (2.5)	0.853
Total lipids in chylomicrons extremely large VLDL	−0.050 (0.303)	0.26 (0.3)	0.22 (0.2)	0.489
Total lipids in very large VLDL	−0.050 (0.299)	0.28 (0.2)	0.26 (0.2)	0.583
Total lipids in large VLDL	−0.046 (0.335)	0.45 (0.2)	0.43 (0.3)	0.613
Total lipids in medium VLDL	−0.033 (0.496)	0.68 (0.2)	0.67 (0.3)	0.869
Total lipids in small VLDL	0.016 (0.739)	0.46 (0.1)	0.47 (0.1)	0.530
Total lipids in very small VLDL	0.135 (0.005)	0.36 (0.1)	0.40 (0.1)	0.011
Total lipids in IDL	0.069 (0.150)	1.18 (0.3)	1.25 (0.3)	0.212
Total lipids in large LDL	−0.013 (0.790)	1.73 (0.4)	1.73 (0.5)	0.988
Total lipids in medium LDL	−0.052 (0.284)	0.77 (0.2)	0.76 (0.2)	0.602
Total lipids in small LDL	−0.038 (0.433)	0.35 (0.1)	0.34 (0.1)	0.843
Total lipids in HDL	0.101 (0.036)	2.96 (0.5)	3.02 (0.6)	0.561
Total lipids in very large HDL	0.278 (<0.001)	0.11 (0.1)	0.15 (0.1)	<0.001
Total lipids in large HDL	0.199 (<0.001)	0.48 (0.2)	0.57 (0.3)	0.027
Total lipids in medium HDL	0.025 (0.601)	1.06 (0.2)	1.04 (0.2)	0.581
Total lipids in small HDL	−0.109 (0.023)	1.31 (0.2)	1.25 (0.1)	0.024
***Total phospholipids in lipoprotein particles***	0.048 (0.320)	2.95 (0.5)	2.99 (0.6)	0.642
Phospholipids in chylomicrons and extremely large VLDL	−0.043 (0.374)	0.03 (0.01)	0.03 (0.02)	0.526
Phospholipids in very large VLDL	−0.038 (0.430)	0.05 (0.03)	0.05 (0.04)	0.737
Phospholipids in large VLDL	−0.042 (0.379)	0.08 (0.05)	0.08 (0.05)	0.678
Phospholipids in medium VLDL	−0.026 (0.590)	0.14 (0.05)	0.14 (0.06)	0.976
Phospholipids in small VLDL	0.011 (0.820)	0.10 (0.03)	0.11 (0.04)	0.571
Phospholipids in very small VLDL	0.143 (0.003)	0.10 (0.02)	0.11 (0.03)	0.007
Phospholipids in IDL	0.067 (0.166)	0.29 (0.07)	0.30 (0.08)	0.228
Phospholipids in large LDL	−0.022 (0.649)	0.39 (0.08)	0.38 (0.09)	0.841
Phospholipids in medium LDL	−0.056 (0.241)	0.19 (0.04)	0.18 (0.05)	0.529
Phospholipids in small LDL	−0.027 (0.576)	0.10 (0.02)	0.09 (0.02)	0.868
Phospholipids in HDL	0.104 (0.031)	1.49 (0.3)	1.52 (0.3)	0.535
Phospholipids in very large HDL	0.288 (<0.001)	0.04 (0.02)	0.06 (0.03)	<0.001
Phospholipids in large HDL	0.200 (<0.001)	0.24 (0.1)	0.28 (0.1)	0.027
Phospholipids in medium HDL	0.026 (0.585)	0.48 (0.1)	0.48 (0.1)	0.612
Phospholipids in small HDL	−0.078 (0.103)	0.72 (0.1)	0.69 (0.1)	0.065
***Total cholesterol***	0.017 (0.724)	5.00 (1.1)	5.06 (1.2)	0.755
Cholesterol in chylomicrons and extremely large VLDL	−0.034 (0.483)	0.06 (0.05)	0.05 (0.04)	0.630
Cholesterol in very large VLDL	−0.035 (0.463)	0.07 (0.04)	0.07 (0.04)	0.776
Cholesterol in large VLDL	−0.033 (0.494)	0.13 (0.07)	0.13 (0.05)	0.811
Cholesterol in medium VLDL	−0.005 (0.919)	0.19 (0.07)	0.19 (0.08)	0.791
Cholesterol in small VLDL	0.030 (0.528)	0.19 (0.06)	0.20 (0.07)	0.339
Cholesterol in very small VLDL	0.129 (0.007)	0.19 (0.06)	0.21 (0.08)	0.016
Cholesterol in IDL	0.059 (0.220)	0.80 (0.2)	0.84 (0.3)	0.286
Cholesterol in large LDL	−0.021 (0.667)	1.24 (0.3)	1.24 (0.3)	0.923
Cholesterol in medium LDL	−0.058 (0.230)	0.55 (0.1)	0.54 (0.2)	0.544
Cholesterol in small LDL	−0.046 (0.343)	0.24 (0.1)	0.23 (0.1)	0.702
Cholesterol in HDL	0.082 (0.089)	1.34 (0.2)	1.36 (0.3)	0.756
Cholesterol in very large HDL	0.253 (<0.001)	0.06 (0.02)	0.08 (0.03)	<0.001
Cholesterol in large HDL	0.186 (<0.001)	0.22 (0.1)	0.26 (0.2)	0.041
Cholesterol in medium HDL	0.011 (0.811)	0.52 (0.1)	0.51 (0.1)	0.438
Cholesterol in small HDL	−0.165 (0.001)	0.54 (0.1)	0.51 (0.1)	0.002
***Triglycerides***	−0.025 (0.597)	1.52 (0.1)	1.49 (0.1)	0.820
Triglycerides in chylomicrons and extremely large VLDL	−0.055 (0.252)	0.17 (0.2)	0.14 (0.1)	0.448
Triglycerides in very large VLDL	−0.058 (0.227)	0.16 (0.1)	0.14 (0.1)	0.484
Triglycerides in large VLDL	−0.053 (0.268)	0.34 (0.1)	0.33 (0.2)	0.515
Triglycerides in medium VLDL	−0.042 (0.383)	0.34 (0.2)	0.33 (0.2)	0.710
Triglycerides in small VLDL	0.003 (0.945)	0.17 (0.1)	0.17 (0.1)	0.787
Triglycerides in very small VLDL	0.101 (0.035)	0.07 (0.02)	0.07 (0.03)	0.066
Triglycerides in IDL	0.134 (0.005)	0.10 (0.02)	0.11 (0.03)	0.018
Triglycerides in large LDL	0.126 (0.008)	0.10 (0.02)	0.11 (0.03)	0.026
Triglycerides in medium LDL	0.075 (0.120)	0.03 (0.01)	0.04 (0.01)	0.173
Triglycerides in small LDL	0.015 (0.754)	0.02 (0.01)	0.02 (0.01)	0.712
Triglycerides in HDL	0.100 (0.037)	0.13 (0.04)	0.14 (0.05)	0.172
Triglycerides in very large HDL	0.146 (0.002)	0.01 (0.002)	0.07 (0.003)	0.017
Triglycerides in large HDL	0.217 (<0.001)	0.02 (0.01)	0.03 (0.02)	0.003
Triglycerides in medium HDL	0.081 (0.092)	0.05 (0.02)	0.05 (0.02)	0.307
Triglycerides in small HDL	0.011 (0.816)	0.05 (0.02)	0.05 (0.02)	0.792
Phosphoglycerides	0.075 (0.120)	2.57 (0.5)	2.64 (0.6)	0.438
Ratio of triglycerides to phosphoglycerides	−0.056 (0.246)	0.57 (0.2)	0.54 (0.2)	0.446
Total cholines	0.073 (0.128)	2.86 (0.5)	2.93 (0.6)	0.450
Phosphatidylcholines	0.073 (0.130)	2.37 (0.5)	2.43 (0.6)	0.489
Sphingomyelins	0.029 (0.545)	0.52 (0.08)	0.52 (0.09)	0.813
Apolipoprotein B	0.004 (0.929)	0.95 (0.3)	0.97 (0.3)	0.705
Apolipoprotein A1	0.091 (0.058)	1.37 (0.2)	1.38 (0.3)	0.698
Ratio of apolipoprotein B to apolipoprotein A1	−0.042 (0.378)	0.70 (0.2)	0.71 (0.2)	0.839
Total fatty acids	0.030 (0.533)	13.60 (2.5)	13.74 (2.9)	0.767
Degree of unsaturation	−0.032 (0.511)	1.30 (0.1)	1.29 (0.1)	0.286
Omega-3 fatty acids	0.029 (0.554)	0.53 (0.1)	0.52 (0.1)	0.842
Omega-6 fatty acids	0.004 (0.942)	4.83 (0.7)	4.79 (0.7)	0.815
Polyunsaturated fatty acids	0.009 (0.857)	5.36 (0.8)	5.32 (0.8)	0.806
Monounsaturated fatty acids	0.036 (0.460)	3.53 (0.9)	3.62 (1.0)	0.582
Saturated fatty acids	0.039 (0.424)	4.72 (0.9)	4.80 (1.2)	0.642
Linoleic acid	0.006 (0.894)	3.73 (0.8)	3.71 (0.8)	0.862
Docosahexaenoic acid	0.037 (0.439)	0.24 (0.05)	0.23 (0.05)	0.384

VLDL, very low density lipoprotein; IDL, intermediate density lipoprotein; LDL, low density lipoprotein; HDL, high density lipoprotein.

### Subgroup analysis showing the effect of lipid lowering medications on lipidomic profile

To demonstrate the impact of anti-lipemic medications on lipid biomolecules, we performed a subgroup analysis between the two groups of patients with T2DM taking versus not taking the lipid lowering agent Supplementary Table S2. Our data revealed that total lipids in lipoprotein particles (*p* = 0.001), total lipids in medium, small, and very small VLDL particles (*p* = 0.021, *p* = 0.045 and *p* = 0.006, respectively), total lipids in large, medium, small LDL particles (*p* < 0.001, *p* = 0.002, and *p* = 0.004, respectively), and total lipids in HDL (*p* < 0.001), very large, large, medium, small HDL particles (*p* = 0.004, *p* = 0.001, *p* = 0.001, and *p* = 0.012 respectively) were significantly lower among patients who were on anti-lipemic medication compared to those who were not on anti-lipemic medication. For total phospholipids in lipoprotein particles (*p* < 0.001), phospholipids in medium, small, very small VLDL particles (*p* = 0.009, *p* = 0.016, and *p* = 0.023, respectively), phospholipids in large, medium, small LDL particles (*p* < 0.001, *p* = 0.002, and *p* = 0.007, respectively), phospholipids in HDL (*p* < 0.001), very large, large, medium, small HDL particles (*p* = 0.005, *p* = 0.001, *p* = 0.003 and *p* = 0.010, respectively) were also significantly lower in patients who were on anti-lipemic medication compared to those who were not on anti-lipemic medication. Additionally, total cholesterol (*p* < 0.001), cholesterol in medium, small, very small VLDL (*p* = 0.001, *p* = 0.012 and *p* = 0.001, respectively), cholesterol in large, medium, and small LDL (*p* < 0.001, *p* = 0.002 and *p* = 0.003, respectively), cholesterol in HDL (*p* < 0.001), very large, large, medium, small HDL (*p* = 0.006, *p* = 0.001, *p* = 0.001 and *p* = 0.020, respectively) were also lower in patients taking anti-lipemic medications. Other lipid biomolecules that were found to be higher in patients not taking anti-lipemic medications compared to those taking these medications were phosphoglycerides (*p* = 0.001), total cholines (*p* < 0.001), phosphatidylcholines (*p* < 0.001), sphingomyelins (*p* < 0.001), Apo B (*p* = 0.001), Apo A (*p* < 0.001), omega 6 fatty acids (*p* = 0.030), PUFA (*p* = 0.007), and linoleic acid (*p* = 0.003).

## Discussion

We have recently demonstrated the prevalence of steatosis and fibrosis, and their associated risk factors in our cohort of patients with T2DM (Alfadda et al., 2022). In our current study of 434 patients with T2DM, CAP values were obtained by FibroScan and alterations in lipidomic profile were demonstrated by using high-throughput proton NMR metabolomics approach. We identified and analyzed a total of 81 lipid biomolecules. Our data highlight an association between steatosis and circulating concentration of lipids, phospholipids, cholesterol and triglycerides in VLDL and LDL subclasses in patients with T2DM. In particular, patients with S3 grade steatosis have higher concentration of lipids, phospholipids, cholesterol and triglycerides in VLDL and LDL subclasses compared to patients with no steatosis. On contrary, a negative association was observed between steatosis and circulating concentration of lipids, phospholipids, cholesterol and triglycerides in HDL subclasses. Moreover, ratio of triglycerides to phosphoglycerides, MUFA and SFA were also significantly higher in patients with S3 grade steatosis compared to patients with no steatosis. Furthermore, an association was observed between fibrosis and concentration of lipids, phospholipids, cholesterol and triglycerides in very small VLDL, large and very large HDL subclasses.

In patients with T2DM, insulin resistance upsurges fatty acid buildup in hepatocytes as a consequence of increased flux of non-esterified fatty acids released during adipose tissue lipolysis and *de novo* lipogenesis (Birkenfeld and Shulman, 2014). These fatty acids are esterified with glycerol to form triglycerides. Increase in the intrahepatic triglyceride accumulation intensifies the formation of lipid droplets in liver (Ress and Kaser, 2016). The triglyceride rich lipids droplets are packaged and secreted as VLDL into circulation and transported to peripheral tissues such as adipose tissue for storage or other metabolic organs like heart, skeletal muscles where they are hydrolyzed to release free fatty acids (FFA) to be consumed for energy (Dowman et al., 2010). The increase in triglyceride buildup and reduction in VLDL secretion and oxidation of fatty acids initiate fat build up in hepatocytes and lead to progression of steatosis (Kawano and Cohen, 2013). In agreement with previous studies, we observed a positive association between steatosis and serum total lipid in VLDL and LDL lipoprotein subclasses, while total lipids HDL subclasses were negatively associated (Kaikkonen et al., 2017). Phospholipids in very large to small subclasses of VLDL were positively associated while very large to large HDL subclasses were negatively associated with steatosis. Furthermore, ANOVA with *post hoc* analysis demonstrated significant differences between total lipids in very large to medium VLDL and very large to large HDL and S0 and S3 grade. Total cholesterol in chylomicrons in very large to large subclasses of VLDL were also significantly different between different grades of steatosis. Additionally, triglycerides and triglycerides in very large to small subclasses of VLDL, medium to small subclasses of LDL and small to medium subclasses of HDL and were significantly different between different grades of steatosis. In consistent with previous studies, cholesterol in very to large subclasses, triglycerides and triglycerides to phosphoglycerides ratio were positively associated with the risk of steatosis (Fukuda et al., 2016). The ratio of triglycerides to phosphoglycerides were significantly higher in S3 grade compared to S0 grade.

The prospective associations of metabolic abnormalities in lipoprotein subclass profile with progression to fatty liver and consequent fibrosis has been revealed previously (Heeren and Scheja, 2021). Recently, an increase in VLDL particle size was linked to steatohepatitis, whereas decrease in the concentration of small VLDL particles was associated with fibrosis (Jiang et al., 2016). In our cohort we observed an association between fibrosis and concentration of lipids and phospholipids of extremely small subclasses of VLDL, small, large and very large subclasses of HDL. A correlation was observed between fibrosis and total cholesterol concentration of very small VLDL and small, large and very large HDL. Additionally, an association was observed between fibrosis and concentration of triglycerides in very small VLDL, IDL, large LDL, HDL, large and very large HDL. A negative correlation but insignificant association was observed between fibrosis and concentration of lipids, phospholipids and cholesterol in small HDL.

The fatty acid composition of the serum reflects the risk of steatosis (Puri et al., 2007). Alteration in the fatty acid composition and dyslipidemia are implicated in the development of early steatosis and NASH (Bjermo et al., 2012; Zheng et al., 2012; Rosqvist et al., 2014; Walle et al., 2016; Luukkonen et al., 2018; Hajduch et al., 2021; Ooi et al., 2021). Serum total MUFA and SFA proportion are reported to be higher in NASH compared to NAFL. Additionally, liver biopsies of patients with steatosis and NASH show increased MUFA and SFA (Puri et al., 2007; Allard et al., 2008; Chiappini et al., 2017). In T2DM patients with NAFLD Increase in circulatory MUFA and SFA are associated with risk of cardiovascular disease (CVD) and strongly with steatosis (Petit et al., 2012; Würtz et al., 2015). In line with these findings, we also observed a positive association between steatosis and serum MUFA and SFA proportion. Our data revealed significant differences in serum MUFA and SFA between patients with no steatosis (S0) and patients with severe steatosis (S3). On the contrary, it has been shown that n‐3 and n‐6 PUFA exhibit a protective role and are inversely related to the steatosis in patients with insulin resistance (López-Vicario et al., 2014). In our study, we observed an association between n‐3 and n‐6 PUFA and steatosis, but no significant differences were observed between different grades of steatosis.

Dyslipidemia or changes in the lipid profile is a common risk factor observed in patients with liver steatosis (Chatrath et al., 2012). Dyslipidemia mainly refers to disorders in lipid metabolism and is characterized by an increase in triglycerides and cholesterol and decrease in HDL in these patients. Thus, use of antilipemic-medications improves lipid profile and decrease the risk of cardiovascular diseases in these patients. For the management of dyslipidemia nearly 47% of the patients with T2DM were on anti-lipemic medication. We looked at the influence of the antilipemic medications in our cohort by performing a sub-analysis of data comparing patients who were on anti-lipemic versus those who were not on anti-lipemic medications. Our data showed a significant decrease in lipids and phospholipids in lipoprotein particles, cholesterol, phosphoglycerides, cholines, phosphatidylcholines, sphingomyelins, ApoB and ApoA1, PUFA and linoleic acid among patients who were on these medications compared to those who were not.

## Conclusion

With the increase in the prevalence of NAFLD in patients with T2DM, there is an urgent need to identify the potential circulation biomarkers to develop an effective screening and therapeutic strategies. In the present study we have utilized a metabolomics platform, and have identified for the first time, the association of circulating adverse lipids and lipoprotein subclasses with NAFLD in Saudi patients with T2DM. Our data demonstrated a significant positive association between steatosis (S3 grade) and dyslipidemia in patients with T2DM. Furthermore, a significant association was observed between liver fibrosis (F2-F4 grade) and concentration of lipids, phospholipids, cholesterol and triglycerides in very small VLDL, large and very large HDL subclasses. On contrary, a negative but weak association was observed between fibrosis and concentration of lipids, phospholipids and cholesterol in small HDL. Furthermore, use of antilipemic medications markedly decreased the concentration of several lipid biomolecules including lipids, phospholipids, and cholesterol in our cohort. In conclusion, the potential serum biomarkers for dyslipidemia identified in current study are important in exploring the association between NAFLD and its associated comorbidities in patients with T2DM.

## Data Availability

The raw data supporting the conclusion of this article will be made available by the authors, without undue reservation.

## Supplementary material

SUPPLEMENTARY TABLE S1 The lipidomic profile of 434 patients with T2DM

SUPPLEMENTARY TABLE S2 Subgroup analysis showing effects of antilipemic medications on lipidomic profile of 434 patients with T2DM
