# Supplementary material for: Alterations in circulating lipidomic profile in patients with type 2 diabetes with or without non-alcoholic fatty liver disease
Source: Front Mol Biosci. 2023 Feb 24;10:1030661. doi: 10.3389/fmolb.2023.1030661 (PMC9999296; doi:10.3389/fmolb.2023.1030661)
Supplement: Supplementary file 1 [file Table1.docx]

**Supplementary Table 1. The lipidomic profile of 434 patients with T2DM**

| **Lipidomic profile** | **Mean (SD)** | **Min-Max** |
| --- | --- | --- |
| ***Total lipids in lipoprotein particles*** | 9.49 (2.10) | 5.48, 20.54 |
| Total lipids in chylomicrons and extremely large VLDL | 0.26 (0.33) | 0, 3.37 |
| Total lipids in very large VLDL | 0.28 (0.21) | 0, 2.13 |
| Total lipids in large VLDL | 0.45 (0.28) | 0.064, 2.81 |
| Total lipids in medium VLDL | 0.67 (0.28) | 0, 2.38 |
| Total lipids in small VLDL | 0.46 (0.16) | 0.18, 1.20 |
| Total lipids in very small VLDL | 0.36 (0.10) | 0.17, 0.82 |
| Total lipids in IDL | 1.19 (0.32) | 0.51, 2.39 |
| Total lipids in large LDL | 1.73 (0.44) | 0.95, 3.43 |
| Total lipids in medium LDL | 0.77 (0.20) | 0.37, 1.58 |
| Total lipids in small LDL | 0.354 (0.08) | 0.2212, 0.69 |
| Total lipids in HDL | 2.97 (0.50) | 1.99, 5.28 |
| Total lipids in very large HDL | 0.11 (0.10) | 0, 0.49 |
| Total lipids in large HDL | 0.49 (0.23) | 0, 1.86 |
| Total lipids in medium HDL | 1.05 (0.19) | 0.62, 1.67 |
| Total lipids in small HDL | 1.31 (0.15) | 0.87, 1.83 |
| ***Total phospholipids in lipoprotein particles*** | 2.95 (0.52) | 1.93, 5.30 |
| Phospholipids in chylomicrons and extremely large VLDL | 0.03 (0.04) | 0, 0.42 |
| Phospholipids in very large VLDL | 0.05 (0.04) | 0, 0.38 |
| Phospholipids in large VLDL | 0.08 (0.05) | 0.01, 0.52 |
| Phospholipids in medium VLDL | 0.14 (0.05) | 0, 0.38 |
| Phospholipids in small VLDL | 0.10 (0.03) | 0.03, 0.25 |
| Phospholipids in very small VLDL | 0.10 (0.03) | 0.04, 0.23 |
| Phospholipids in IDL | 0.29 (0.07) | 0.12, 0.55 |
| Phospholipids in large LDL | 0.39 (0.09) | 0.22, 0.72 |
| Phospholipids in medium LDL | 0.19 (0.04) | 0.09, 0.36 |
| Phospholipids in small LDL | 0.09 (0.02) | 0.06, 0.18 |
| Phospholipids in HDL | 1.49 (0.26) | 0.98, 2.68 |
| Phospholipids in very large HDL | 0.05 (0.03) | 0, 0.26 |
| Phospholipids in large HDL | 0.24 (0.11) | 0, 0.89 |
| Phospholipids in medium HDL | 0.48 (0.09) | 0.28, 0.79 |
| Phospholipids in small HDL | 0.72 (0.09) | 0.46, 1.09 |
| ***Total cholesterol*** | 5.01 (1.1) | 2.96, 9.38 |
| Cholesterol in chylomicrons and extremely large VLDL | 0.054 (0.065) | 0, 0.6494 |
| Cholesterol in very large VLDL | 0.073 (0.045) | 0, 0.4312 |
| Cholesterol in large VLDL | 0.130 (0.07) | 0.0159, 0.6586 |
| Cholesterol in medium VLDL | 0.195 (0.08) | 0, 0.5303 |
| Cholesterol in small VLDL | 0.1954 (0.063) | 0.0823, 0.4812 |
| Cholesterol in very small VLDL | 0.1976 (0.06) | 0.0965, 0.4390 |
| Cholesterol in IDL | 0.814 (0.23) | 0.2932, 1.651 |
| Cholesterol in large LDL | 1.27 (0.34) | 0.66, 2.51 |
| Cholesterol in medium LDL | 0.564 (0.15) | 0.148, 0.3089 |
| Cholesterol in small LDL | 0.241 (0.06) | 0.1444, 0.4748 |
| Cholesterol in HDL | 1.34 (0.2) | 0.74, 2.34 |
| Cholesterol in very large HDL | 0.063 (0.02) | 0.021, 0.2161 |
| Cholesterol in large HDL | 0.218 (0.113) | 0.0048, 0.8820 |
| Cholesterol in medium HDL | 0.52 (0.09) | 0.2915, 0.8093 |
| Cholesterol in small HDL | 0.54 (0.06) | 0.35, 0.68 |
| ***Triglycerides*** | 1.52 (0.8) | 0.39, 8.87 |
| Triglycerides in chylomicrons and extremely large VLDL | 0.17 (0.227) | 0, 0.230 |
| Triglycerides in very large VLDL | 0.159 (0.130) | 0, 1.32 |
| Triglycerides in large VLDL | 0.237 (0.160) | 0.034, 1.621 |
| Triglycerides in medium VLDL | 0.341 (0.174) | 0, 1.725 |
| Triglycerides in small VLDL | 0.1756 (0.07) | 0.069, 0.6872 |
| Triglycerides in very small VLDL | 0.071 (0.022) | 0.0266, 0.1914 |
| Triglycerides in IDL | 0.102 (0.03) | 0.048, 0.219 |
| Triglycerides in large LDL | 0.101 (0.03) | 0.0499, 0.2153 |
| Triglycerides in medium LDL | 0.035 (0.01) | 0.0152, 0.0966 |
| Triglycerides in small LDL | 0.016 (0.006) | 0.0064, 0.0640 |
| Triglycerides in HDL | 0.13 (0.04) | 0.05, 0.44 |
| Triglycerides in very large HDL | 0.006 (0.002) | 0.0015, 0.0257 |
| Triglycerides in large HDL | 0.022 (0.01) | 0.0036, 0.0829 |
| Triglycerides in medium HDL | 0.05 (0.02) | 0.015, 0.1676 |
| Triglycerides in small HDL | 0.06 (0.02) | 0.02, 0.17 |
| Phosphoglycerides | 2.58 (0.50) | 1.51, 4.40 |
| Ratio of triglycerides to phosphoglycerides | 0.57 (0.22) | 0.22, 1.51 |
| Total cholines | 2.86 (0.51) | 1.79, 4.77 |
| Phosphatidylcholines | 2.37 (0.5) | 1.29, 4.18 |
| Sphingomyelins | 0.52 (0.08) | 0.32, 0.80 |
| Apolipoprotein B | 0.95 (0.3) | 0.42, 2.18 |
| Apolipoprotein A1 | 1.37 (0.2) | 0.92, 2.37 |
| Ratio of apolipoprotein B to apolipoprotein A1 | 0.70 (0.2) | 0.26, 1.59 |
| Total fatty acids | 13.62 (2.6) | 8.80, 23.83 |
| Degree of unsaturation | 1.31 (0.09) | 1.03, 1.53 |
| Omega-3 fatty acids | 0.53 (0.2) | 0.22, 1.18 |
| Omega-6 fatty acids | 4.82 (0.7) | 3.48, 8.10 |
| Polyunsaturated fatty acids | 5.35 (0.8) | 3.72, 8.78 |
| Monounsaturated fatty acids | 3.54 (0.9) | 2.05, 8.04 |
| Saturated fatty acids | 4.72 (0.9) | 3.02, 9.05 |
| Linoleic acid | 3.73 (0.8) | 2.28, 7.44 |
| Docosahexaenoic acid | 0.24 (0.05) | 0.11, 0.52 |

VLDL – very low-density lipoprotein; IDL – intermediate density lipoprotein; LDL – low density lipoprotein; HDL – high density lipoprotein

**Supplementary Table 2: Subgroup analysis showing effects of antilipemic medications on lipidomic profile of 434 patients with T2DM**

| Biomolecules | On antilipemic medication  N=203 | Not on antilipemic medication  N=231 | P values |
| --- | --- | --- | --- |
| **Total lipids in lipoprotein particles** | 9.12 (1.9) | 9.81 (2.2) | 0.001 |
| Total lipids in chylomicrons extremely large VLDL (XXL-VLDL-L) | 0.25 (0.3) | 0.26 (0.3) | 0.850 |
| Total lipids in very large VLDL (XL-VLDL-L) | 0.27 (0.2) | 0.29 (0.2) | 0.548 |
| Total lipids in large VLDL (L-VLDL-L) | 0.44 (0.2) | 0.46 (0.3) | 0.427 |
| Total lipids in medium VLDL (M-VLDL-L) | 0.64 (0.3) | 0.70 (0.3) | 0.021 |
| Total lipids in small VLDL (S-VLDL-L) | 0.44 (0.1) | 0.47 (0.2) | 0.045 |
| Total lipids in very small VLDL (XS-VLDL-L) | 0.35 (0.1) | 0.37 (0.1) | 0.006 |
| Total lipids in IDL (IDL-L) | 1.1 (0.3) | 1.2 (0.3) | <0.001 |
| Total lipids in large LDL (L-LDL-L) | 1.65 (0.4) | 1.80 (0.4) | <0.001 |
| Total lipids in medium LDL (M-LDL-L) | 0.74 (0.2) | 0.80 (0.2) | 0.002 |
| Total lipids in small LDL (S-LDL-L) | 0.33 (0.1) | 0.36 (0.1) | 0.004 |
| Total lipids in HDL (HDL-L) | 2.87 (0.4) | 3.06 (0.6) | <0.001 |
| Total lipids in very large HDL (XL-HDL-L) | 1.0 (0.1) | 0.12 (0.1) | 0.004 |
| Total lipids in large HDL (L-HDL-L) | 0.45 (0.2) | 0.50 (0.3) | 0.001 |
| Total lipids in medium HDL (M-HDL-L) | 1.02 (0.2) | 1.08 (0.2) | 0.001 |
| Total lipids in small HDL (S-HDL-L) | 1.30 (0.1) | 1.30 (0.1) | 0.012 |
| **Total phospholipids in lipoprotein particles** | 2.85 (0.5) | 3.05 (0.5) | <0.001 |
| Phospholipids in chylomicrons and extremely large VLDL (XXL-VLDL-PL) | 0.03 (0.04) | 0.03 (0.04) | 0.864 |
| Phospholipids in very large VLDL (XL-VLDL-PL) | 0.05 (0.04) | 0.05 (0.04) | 0.605 |
| Phospholipids in large VLDL (L-VLDL-PL) | 0.08 (0.05) | 0.09 (0.06) | 0.522 |
| Phospholipids in medium VLDL (M-VLDL-PL) | 0.13 (0.05) | 0.14 (0.05) | 0.009 |
| Phospholipids in small VLDL (S-VLDL-PL) | 0.09 (0.03) | 0.11 (0.03) | 0.016 |
| Phospholipids in very small VLDL (XS-VLDL-PL) | 0.09 (0.03) | 0.10 (0.03) | 0.023 |
| Phospholipids in IDL (IDL-PL) | 0.27 (0.1) | 0.30 (0.1) | <0.001 |
| Phospholipids in large LDL (L-LDL-PL) | 0.37 (0.1) | 0.40 (0.1) | <0.001 |
| Phospholipids in medium LDL (M-LDL-PL) | 0.18 (0.04) | 0.20 (0.1) | 0.002 |
| Phospholipids in small LDL (S-LDL-PL) | 0.09 (0.02) | 0.10 (0.02) | 0.007 |
| Phospholipids in HDL (HDL-PL) | 1.44 (0.2) | 1.50 (0.3) | <0.001 |
| Phospholipids in very large HDL (XL-HDL-PL) | 0.04 (0.02) | 0.04 (0.04) | 0.005 |
| Phospholipids in large HDL (L-HDL-PL) | 0.23 (0.1) | 0.26 (0.1) | 0.001 |
| Phospholipids in medium HDL (M-HDL-PL) | 0.47 (0.1) | 0.48 (0.1) | 0.003 |
| Phospholipids in small HDL (S-HDL-PL) | 0.71 (0.1) | 0.72 (0.1) | 0.010 |
| **Total cholesterol** | 4.78 (1.0) | 5.21 (1.1) | <0.001 |
| Cholesterol in chylomicrons and extremely large VLDL (XXL-VLDL-C) | 0.05 (0.1) | 0.06 (0.1) | 0.711 |
| Cholesterol in very large VLDL (XL-VLDL-C) | 0.10 (0.04) | 0.10 (0.05) | 0.304 |
| Cholesterol in large VLDL (L-VLDL-C) | 0.13 (0.1) | 0.13 (0.1) | 0.266 |
| Cholesterol in medium VLDL (M-VLDL-C) | 0.18 (0.1) | 0.20 (0.1) | 0.001 |
| Cholesterol in small VLDL (S-VLDL-C) | 0.18 (0.1) | 0.20 (0.04) | 0.012 |
| Cholesterol in very small VLDL (XS-VLDL-C) | 0.18 (0.1) | 0.20 (0.05) | 0.001 |
| Cholesterol in IDL (IDL-C) | 0.76 (0.2) | 0.84 (0.2) | <0.001 |
| Cholesterol in large LDL (L-LDL-C) | 1.18 (0.3) | 1.29 (0.3) | <0.001 |
| Cholesterol in medium LDL (M-LDL-C) | 0.53 (0.1) | 0.57 (0.1) | 0.002 |
| Cholesterol in small LDL (S-LDL-C) | 0.23 (0.1) | 0.24 (0.1) | 0.003 |
| Cholesterol in HDL (HDL-C) | 1.30 (0.2) | 1.39 (0.3) | <0.001 |
| Cholesterol in very large HDL (XL-HDL-C) | 0.06 (0.02) | 0.07 (0.03) | 0.006 |
| Cholesterol in large HDL (L-HDL-C) | 0.21 (0.1) | 0.24 (0.1) | 0.001 |
| Cholesterol in medium HDL (M-HDL-C) | 0.52 (0.04) | 0.53 (0.1) | 0.001 |
| Cholesterol in small HDL (S-HDL-C) | 0.53 (0.1) | 0.54 (0.1) | 0.020 |
| **Triglycerides** | 1.49 (0.8) | 1.55 (0.9) | 0.404 |
| Triglycerides in chylomicrons and extremely large VLDL (XXL-VLDL-TG) | 0.17 (0.2) | 0.17 (0.2) | 0.889 |
| Triglycerides in very large VLDL (XL-VLDL-TG) | 0.15 (0.1) | 0.16 (1.3) | 0.637 |
| Triglycerides in large VLDL (L-VLDL-TG) | 0.23 (0.1) | 0.24 (0.1) | 0.488 |
| Triglycerides in medium VLDL (M-VLDL-TG) | 0.33 (0.2) | 0.35 (0.2) | 0.171 |
| Triglycerides in small VLDL (S-VLDL-TG) | 0.16 (0.1) | 0.17 (0.1) | 0.294 |
| Triglycerides in very small VLDL (XS-VLDL-TG) | 0.07 (0.02) | 0.17 (0.1) | 0.212 |
| Triglycerides in IDL (IDL-TG) | 0.10 (0.02) | 0.10 (0.03) | 0.065 |
| Triglycerides in large LDL (L-LDL-TG) | 0.09 (0.02) | 0.10 (0.03) | 0.042 |
| Triglycerides in medium LDL (M-LDL-TG) | 0.03 (0.01) | 0.03 (0.01) | 0.089 |
| Triglycerides in small LDL (S-LDL-TG) | 0.01 (0.01) | 0.02 (0.01) | 0.295 |
| Triglycerides in HDL (HDL-TG) | 0.13 (0.03) | 0.13 (0.05) | 0.078 |
| Triglycerides in very large HDL (XL-HDL-TG) | 0.01 (0.01) | 0.01 (0.03) | 0.027 |
| Triglycerides in large HDL (L-HDL-TG) | 0.02 (0.01) | 0.02 (0.02) | 0.002 |
| Triglycerides in medium HDL (M-HDL-TG) | 0.05 (0.01) | 0.05 (0.01) | 0.101 |
| Triglycerides in small HDL (S-HDL-TG) | 0.05 (0.01) | 0.05 (0.02) | 0.447 |
| Phosphoglyccerides | 2.49 (0.5) | 2.66 (0.5) | 0.001 |
| Ratio of triglycerides to phosphoglycerides (TG/PG) | 0.58 (0.2) | 0.56 (0.2) | 0.388 |
| Total cholines | 2.77 (0.5) | 2.96 (0.5) | <0.001 |
| Phosphatidylcholines | 2.29 (0.4) | 2.45 (0.5) | <0.001 |
| Sphingomyelins | 0.50 (0.1) | 0.54 (0.1) | <0.001 |
| Apolipoprotein B (Apo B) | 0.91 (0.3) | 0.99 (0.3) | 0.001 |
| Apolipoprotein A1 (Apo A1) | 1.33 (0.2) | 1.41 (0.2) | <0.001 |
| Ratio of apolipoprotein B to apolipoprotein A1 (Apo B/Apo A1) | 0.69 (0.2) | 0.72 (0.2) | 0.124 |
| Total fatty acids (TFA) | 13.36 (2.5) | 13.84 (2.6) | 0.054 |
| Degree of unsaturation | 1.29 (0.1) | 1.31 (0.1) | 0.030 |
| Omega-3 fatty acids | 0.53 (0.2) | 0.53 (0.1) | 0.864 |
| Omega-6 fatty acids | 4.72 (0.6) | 4.92 (0.7) | 0.030 |
| Polyunsaturated fatty acids (PUFA) | 5.24 (0.7) | 5.45 (0.8) | 0.007 |
| Monounsaturated fatty acids (MUFA) | 3.48 (0.9) | 3.59 (0.9) | 0.212 |
| Saturated fatty acids (SFA) | 4.64 (1.0) | 4.80 (0.9) | 0.091 |
| Linoleic acid (LA) | 3.61 (0.7) | 3.84 (0.8) | 0.003 |
| Docosahexaenoic acid (DHA) | 0.23 (0.04) | 0.24 (0.05) | 0.053 |

VLDL – very low-density lipoprotein; IDL – intermediate density lipoprotein; LDL – low density lipoprotein; HDL – high density lipoprotein
